# The Importance of Recognizing Wandering Spleen as a Cause of Recurrent Acute Pancreatitis

**DOI:** 10.1155/2018/7573835

**Published:** 2018-06-21

**Authors:** Vennis Lourdusamy, Dhrumil Patel, Ramon Docobo, Shyamanand Tantry, Dennis Lourdusamy, Saeed Farukh

**Affiliations:** ^1^Department of Internal Medicine, Brandon Regional Hospital, Brandon, Florida 33511, USA; ^2^Department of Internal Medicine, Trinitas Regional Medical Center, Elizabeth, New Jersey 07202, USA; ^3^Department of Gastroenterology and Hepatology, Brandon Regional Hospital, Brandon, Florida 33511, USA

## Abstract

Wandering spleen, as its name suggests, is a rare condition where the spleen wanders in the abdomen and is most commonly found in the inferior quadrant as a palpable mass. It can have varying presentations and commonly presents as splenic torsion and very rarely as acute pancreatitis. It is imperative not to miss this diagnosis as it can lead to life-threatening complications in the form of splenic torsion/infarction. Here we describe a rare manifestation of wandering spleen where a young female presented with recurrent episodes of acute pancreatitis.

## 1. Introduction

Wandering spleen is a rare condition where the spleen is not found in its normal location. It occurs secondary to developmental abnormality or acquired laxity of the ligaments that hold the spleen resulting in abnormal location of the spleen and hence affects the children and young adults especially women of child bearing age [1]. It can be difficult to diagnose this condition as the symptoms can be nonspecific and always requires a high degree of suspicion.

## 2. Case Presentation

A 28-year-old multiparous female presented to the Emergency Department with complaints of mild abdominal pain associated with nausea for two days. She described her pain episodes as mild in nature, located in the epigastric/left upper quadrant, nonradiating, and slight worsening with food intake. She denied fever, chills, vomiting, or diarrhea.

Her past medical history was significant for a similar presentation about 6 months before when she was diagnosed with idiopathic acute pancreatitis. She denied any alcohol intake and was not on any medications or herbal supplements.

**Figure 1:**
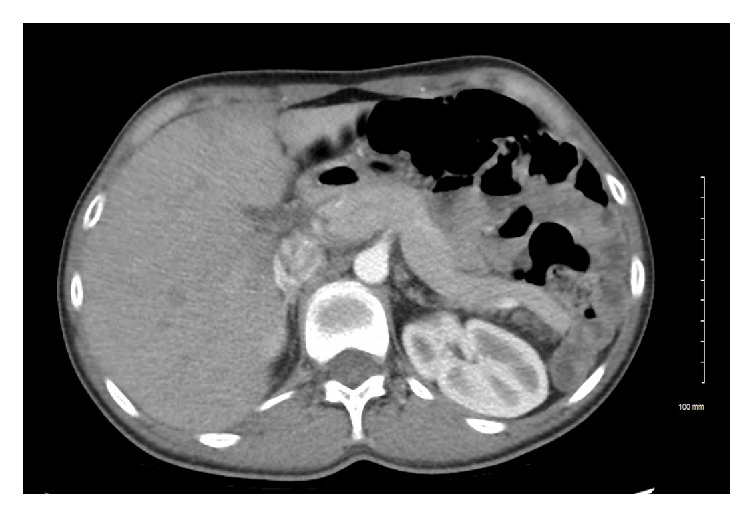
Axial view CT of the abdomen showing elongated pancreatic tail.

**Figure 2:**
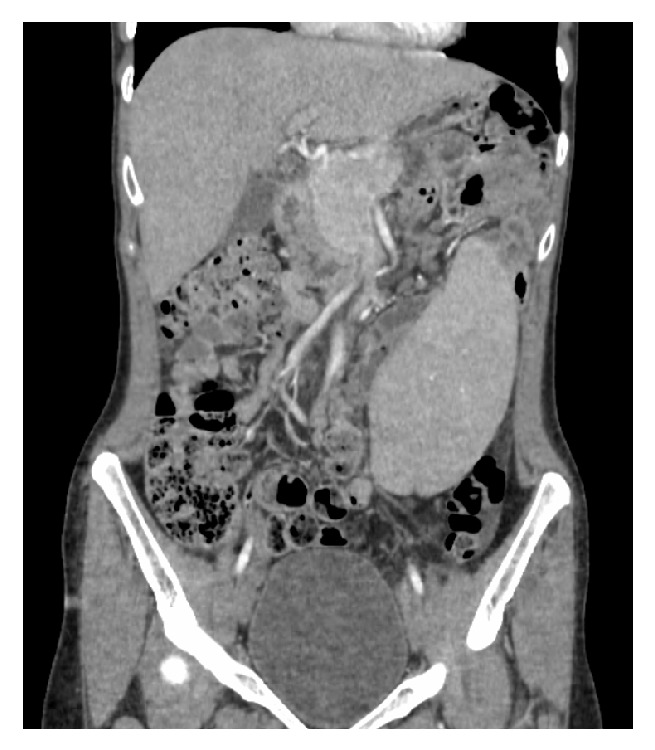
CT of the abdomen demonstrating spleen in the inferior quadrant of the abdomen.

On admission, she was afebrile with a pulse rate of 84 beats per minute and a blood pressure of 116/80 mm Hg. Examination of the abdomen revealed mild tenderness in the epigastric region/left upper quadrant region and also with a palpable mass in the left lower quadrant. Laboratory workup revealed elevated lipase (4337), unremarkable CBC, and, with normal liver functions tests, lipid panel and IgG panel. Ultrasound of abdomen showed minimal sludge in the gall bladder without any obvious stones. CT abdomen with contrast demonstrated a spleen in the anterior left lower abdomen, elongated pancreatic tail which was coiled in conjunction with the splenic vessels, and with mild inflammation of the pancreatic tail (Figures 1 and 2). She improved clinically with conservative management with IV fluids. She finally underwent splenopexy at an outside facility.

## 3. Discussion

Wandering spleen is a rare finding, and only a few case reports of this condition causing recurrent abdominal pain secondary to pancreatitis have been published in literature. Our patient was diagnosed with this condition after her second episode of acute pancreatitis in a span of 6 months, and she eventually underwent splenopexy to prevent further complications.

The presentation is variable, and it commonly presents as splenic torsion which will be described below. A few cases are incidentally diagnosed with a palpable spleen on physical examination as the spleen lies inferiorly, and presentation can be asymptomatic if diagnosed before the occurrence of complications. It can present as recurrent abdominal pain secondary to torsion and spontaneous detorsion of the splenic pedicle [2]. Acute pancreatitis in this setting usually involves the inflammation of the tail of the pancreas likely in the setting of intermittent torsion of the vascular pedicle at the splenic hilum along with the tail of the pancreas which is also a part of the hilum [3]. Congenital form of wandering spleen occurs secondary to the incomplete fusion of the dorsal mesogastrium in which the spleen, body, and tail of the pancreas and the splenorenal ligament develop from [4]. The increased laxity of the spleen results in the elongation of the vascular pedicle which predisposes it to torsion [5]. Although our patient did not present with signs of splenic torsion, the imaging finding was consistent with elongation of the pancreatic tail in conjunction with splenic vessels. Our patient probably had episodes of intermittent torsion of the splenic hilar structures which caused her recurrent pancreatitis. Splenic torsion is a life-threatening condition which can result in splenic infarction presenting as acute abdomen and needs surgical management mainly in the form of splenectomy [6]. Intermittent torsions can also result in splenomegaly secondary to venous congestion with features of hypersplenism. Other rare presentation includes upper GI bleeding secondary to gastric varices from chronic torsion of the vascular pedicle (splenic vein occlusion resulting in left sided portal hypertension) or rarely in the setting of splenic vein thrombosis [7, 8].

Imaging findings including ultrasound or CT of the abdomen play an important role in confirming the diagnosis as the presentation of this condition is variable and ruling out splenic torsion. Ultrasound of the abdomen will locate the abnormal position of the spleen, and Doppler flow should be done in those cases to demonstrate the blood flow in the splenic vessels. The diagnosis can be complicated in certain cases when spleen can be located in the normal position at the time of the imaging especially if the imaging is delayed [9]. This is secondary to the intermittent nature of torsion/wandering of the spleen and is usually diagnosed during subsequent episodes of pancreatitis [3]. Hence high degree of suspicion for this condition is the key to diagnosis. CT abdomen with contrast during her second episode of pancreatitis established the diagnosis in our patient with abnormal location of the spleen in the anteroinferior quadrant along with the elongation of the pancreatic tail in conjunction with splenic vessels.

Management depends on the presentation with splenectomy reserved for those with evidence of splenic infarction secondary to torsion. Splenopexy with splenic preservation is usually the choice for young patients without significant splenomegaly or evidence of splenic infarction as in our patient who eventually underwent elective splenopexy to fix the spleen in the normal location after the initial conservative management of acute pancreatitis.

## 4. Conclusion

Wandering spleen as a cause of pancreatitis should be considered especially in children and young females who present with recurrent pancreatitis and without obvious causes for pancreatitis. It is a very rare occurrence and probably subclinical in many cases as the inflammation only involves the tail of the pancreas and hence many might not seek medical attention. This probably explains the rarity of literature published on pancreatitis in the setting of wandering spleen as opposed to that of splenic torsion/infarction. Nevertheless, the importance of recognition of this condition in those presenting with acute pancreatitis is paramount to prevent acute life-threatening complications in future with early surgical intervention.
